# Relationship between VEGF Family Members, Their Receptors and Cell Death in the Neoplastic Transformation of Colorectal Cancer

**DOI:** 10.3390/ijms23063375

**Published:** 2022-03-21

**Authors:** Dominika Dakowicz, Monika Zajkowska, Barbara Mroczko

**Affiliations:** 1 Department of Neurodegeneration Diagnostics, Medical University of Bialystok, 15-269 Bialystok, Poland; dominika.dakowicz@sd.umb.edu.pl (D.D.); mroczko@umb.edu.pl (B.M.); 2 Department of Biochemical Diagnostics, Medical University of Bialystok, 15-269 Bialystok, Poland

**Keywords:** CRC, VEGF, apoptosis, diagnosis, tumor markers, gastrointestinal tract tumors, angiogenesis

## Abstract

Colorectal cancer (CRC) is the second most common cause of cancer death in the world. Both modifiable and nonmodifiable risk factors play a significant role in the pathogenesis of this tumor. The diagnosis is usually made late due to limitations of screening tests; therefore, the scientists are looking for new diagnostic tools such as gene or miRNA expression or different proteins’ concentrations, e.g., vascular endothelial growth factor (VEGF) family members. The VEGF family (VEGF-A, VEGF-B, VEGF-C, VEGF-D and PlGF) plays a key role in the processes of blood vessel formation in embryonic development as well as in pathological angiogenesis and lymphangiogenesis, which allow the tumor to grow exponentially. Blockage of VEGF-related pathways seems to be a valid therapeutic target. It was suggested in recent studies, that besides already used drugs, e.g., bevacizumab, there are other agents with potential usefulness in anticancer activity such as miRNAs, TMEA, granzyme K, baicalein and arginine. Moreover, VEGF proteins were assessed to induce the expression of anti-apoptotic proteins such as BCL-2 and BAX. Therefore, investigations concerning the usefulness of VEGF family members, not only in the development but also in the therapy of CRC, in order to fully elucidate their role in carcinogenesis, are extremely important.

## 1. Colorectal Cancer

### 1.1. Epidemiology

Colorectal cancer (CRC) is the second most common cause of cancer death in the world and the third in terms of incidence. Geographically, the highest rates of occurrence of CRC are in Europe, Australia and Northern America. The incidence of CRC is strongly related to economic status; therefore, this tumor is more common in developed than in developing countries due to economic progress that results in an increase in average life expectancy and population growth. Risk factors of colorectal cancer can be divided into modifiable and nonmodifiable ones. Nonmodifiable risk factors include male sex, due to global incidence of sexual dimorphism in CRC rates that is related to the protective role of estrogen against colorectal cancer development; age; familial history of colorectal polyps or CRC; personal history of Crohn’s disease or ulcerative colitis; hereditary conditions such as Lynch syndrome, familial adenomatous polyposis (FAP) and MUTYH-associated polyposis (MAP); racial and ethnic backgrounds and the presence of type 2 diabetes. Recent studies also revealed that being tall increases the risk of colorectal cancer. Modifiable risk factors include adoption of the ‘Western lifestyle’ comprehended as sedentary lifestyle that leads to an increase in excess body weight and eating habits characterized by a higher intake of red meat, processed meat, fat and calories, as well as alcohol consumption and cigarette smoking. Other associations with CRC include history of abdominal radiation, renal transplant followed by immunosuppression, insulin resistance, coronary artery disease, cholecystectomy and acromegaly. On the other hand, there is strong evidence that calcium supplements and adequate consumption of fiber, whole grains and dairy products decrease the risk of colorectal cancer. Interestingly, regular physical activity and consumption of fish; garlic; foods with vitamin C, vitamin D, vitamin B6; curcumin also called the golden spice and multivitamin supplements, as well as regular use of aspirin and non-steroidal anti-inflammatory drugs (NSAIDs) appear to decrease the risk, although further investigations are necessary [1,2,3,4,5,6,7].

### 1.2. Pathogenesis

The precise pathogenesis of colorectal cancer remains unclear; however, there have been four main hypotheses that have been investigated throughout the years. First of all, genetic and epigenetic changes increase the risk of developing colorectal cancer. Second, cancer develops based on a precancerous condition that progresses into the malignant stage. Third, development of cancer is due to lack of genetic stability characterized by subchromosomal genomic amplifications and chromosome imbalances. Fourth, the hereditary aspect of colorectal cancer might play a significant role, especially in terms of rare colon cancers [8,9].

Regardless of which hypothesis was considered, it has been shown that colon cancer develops as a result of uncontrolled cell growth. It is characterized by an insidious and asymptomatic course, and the first symptoms of the disease usually appear when the cancer process is in an advanced stage. In the case of this tumor, early diagnosis is crucial. Intensive proliferation of cancer cells, increasing with the stage of the cancer, requires an increased supply of oxygen and nutrients; therefore, in the development of colorectal cancer, there is an intensification of the processes of angiogenesis and lymphangiogenesis, leading to the formation of new blood and lymphatic vessels. The blood vessels supply oxygen and nourishment to tissues and eliminate metabolic products and carbon dioxide, which results in intensive tissue growth. One of the most important stimulators of these mechanisms are proteins from the VEGF family (vascular endothelial growth factors) that enable these processes [10,11,12].

Some studies also reveal a correlation between colorectal cancer and the role of gut microbiota. It has been indicated that pro-inflammatory bacteria such as *Fusobacterium nucleatum* simulates local inflammatory response and reduces immune reactions. Moreover, *Fusobacterium nucleatum* has demonstrated a positive correlation with lymph node metastasis in CRC. These bacteria have been observed also as activating factors to the wingless/integrated-1 (WNT) signaling pathway and their impact on epigenetic silencing of MMR Protein, MLH1, which may result in microsatellite-instable CRC [5,13].

### 1.3. Classification

In order to assess the stage of the cancer and, thus, provide accurate prognostic information and select the most adequate therapies that will affect the future of the patient, the cancer should be properly classified. Thus far, several scales are available, including the most important TNM scale as well as histopathological differentiation and the Dukes’ scale, which has more historical background. The TNM classification stands for T—tumor, N—lymph node, M—metastasis and is widely used, and most therapeutic decisions have been made based on that scale. Currently there is the eighth edition of the AJCC TNM classification (Table 1). It is also important to identify the tumor according to its histopathological properties: i.e., as adenocarcinoma, adenocarcinoma mucinosum, adenocarcinoma mucocellulare, carcinoma planoepitheliale. The Dukes’ scale was firstly established in 1958 and consisted of four stages: A, B, C and D, which identified groups of patients with different prognosis. A—tumor confined to the intestinal wall, B—the tumor crosses the intestinal wall, C—metastases in the lymph nodes, D—distant metastases [14,15].

### 1.4. Diagnostics

Currently, the diagnostics of colorectal cancer are limited to screening tests, among which colonoscopy remains the study of choice to diagnose colorectal cancer followed by sigmoidoscopy and fecal occult blood testing (FOBT). Unfortunately, screening effectiveness is restricted by limitations of test performance. Therefore, it is important to establish new biomarkers that will have high diagnostic sensitivity and specificity in detecting colorectal cancer at the early stage. Consequently, researchers are searching for different, new parameters to find markers for highly accurate and non-invasive screening tests for colorectal cancer. Thus far, there are some parameters that have been studied for their potential use in CRC screening: e.g., carcinoembryonic antigen (CEA); cancer antigen 19-9 (CA 19-9); DNA, e.g., SMAD7 gene; proteins; messenger RNA (mRNA); microRNA (miRNA) and vascular endothelial growth factor (VEGF) family members.

Currently, CEA levels are used as a tool for follow-up observation of patients after treatment rather than to diagnose the tumor itself [1,2,3,5,16,17]. A rapid increase in CEA concentration after surgical resection suggests the occurrence of metastases; a slowly increasing level is typical sign of local recurrence [18]. The literature also reveals that an elevated alkaline phosphatase (ALP) level is the most common altered laboratory parameter in patients with liver metastases. Nevertheless, it is assumed that magnetic resonance imaging (MRI) of the liver is more accurate in its detection [5]. Recent research shows that an elevated level of ezrin protein might play a significant role in the tumor invasion process. It has been found that overexpression of ezrin protein correlates with worse prognosis and higher aggressiveness of colorectal cancer. High ezrin expression might also be a marker of local recurrence of rectal cancer [18].

Another diagnostic path involves the detection of circulating tumor DNA (ctDNA) released into the circulation from tumor cells after apoptosis or necrosis. ctDNA carries tumor genetic or epigenetic alterations. Analysis of ctDNA might potentially be a useful diagnostic tool due to relatively easy access to the material via blood samples [19]. It has also been presented that overexpression of LBX2, a transcription factor gene that is located on chromosome 2p13.1, is associated with colorectal cancer progression and poor prognosis. The abnormal expression of LBX2 has been observed in other types of cancers, such as non-small-cell lung cancer, adenoid cystic carcinoma and T-cell acute lymphoblastic leukemia [20]. Recently, studies have found that the expression of lncRNA-LOC100127888 (LncA), a long noncoding RNA, is upregulated in colorectal cancer tissues and correlated with TNM stage, pathological classification and lymph node and distant metastasis. LncA is suggested to potentially be a novel biomarker to predict the prognosis of colorectal cancer disease [21]. Moreover, some scientists have recently investigated the significance of a novel biomarker, cholesterol-to-lymphocyte ratio (CLR), and the conclusions claim that it can be a potential tool in predicting survival in patients with CRC [8].

As early diagnosis and determination of cancer advancement allows to increase the survival rate of patients with CRC by identifying effective treatment methods, it is important to constantly search for new prognostic and diagnostic factors. These markers can enable early detection and surgical removal of the neoplastic tumor, and at the same time, could allow, if necessary, the use of other available treatment options such as chemotherapy or radiotherapy. The processes that significantly contribute to the growth of neoplastic lesions and their metastasis are angio- and lymphangiogenesis, and the substances having the greatest influence on these processes are factors from the VEGF family and their receptors. Therefore, studies confirming their effectiveness as tumor markers are needed not only in tumor detection but also in determining and monitoring subsequent treatment. Introducing early diagnosis and effective therapy becomes the mission of modern health protection, especially in the case of a common disease such as CRC.

## 2. VEGF Family and Their Receptors

**Figure 1:**
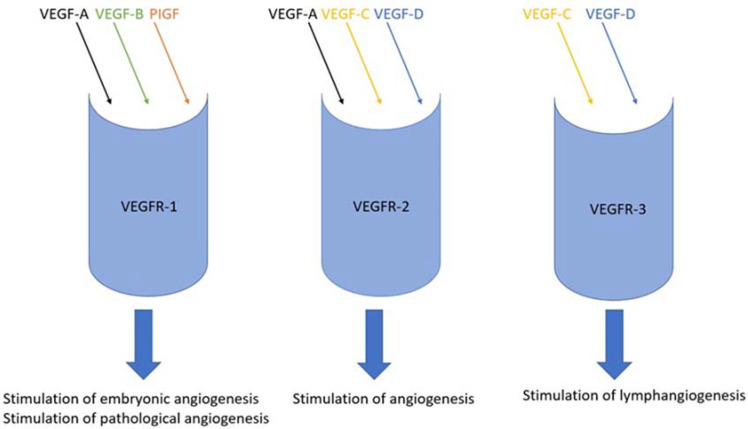
The possible combinations of bindings of VEGF family factors to individual receptors.

The VEGF family comprises a group of proteins called growth factors. They are part of a wider set of signaling proteins—cytokines. This subgroup contains five proteins (VEGF-A, VEGF-B, VEGF-C, VEGF-D and PlGF (placental growth factor)) that have a significant impact on angio- and lymphangiogenesis processes [11,12]. It has been identified that expression of VEGF proteins is correlated to hypoxia [22]. Hypoxia might result in an elevated effector state that involves the production of IFNγ and increased cytolytic activity. This elevated effector state relates to hypoxia-induced stabilization of HIF1α, which is the transcription factor that regulates many processes, e.g., angiogenesis, glycolysis, cell survival and others [23]. There are three commonly known receptors for agents of the VEGF family (VEGFR-1, VEGFR-2 and VEGFR-3). Each of them has the opportunity to combine selected factors belonging to the VEGF family because of different affinity and selectivity. These factors can be divided based on their biological activities (Figure 1) [11,12]. VEGF-A, VEGF-B and PlGF have different isoforms, while VEGF-C and VEGF-D are proteolytically processed to mature form. VEGF-A creates isoforms and four of them occur in humans—VEGF165, VEGF121, VEGF189 and VEGF206. From the point of view of quantity and biological activity, the dominating form among them is VEGF165. It is overexpressed in many cancers and is often correlated with progression [11].

In humans, the VEGF-A (also called VEGF) gene at locus 6p21.3 is part of a group of genes that encode a cluster of proteins called the cystine-knot growth factor superfamily, which also includes PDGF, NGF and TGF-β. VEGF is a glycoprotein with a heterodimer structure in which the cystine node has a characteristic arrangement of disulfide bridges. They are secreted physiologically but also by tumor cells and the tissues surrounding the tumor in a hypoxic state, and subsequently, they bind to kinase-like receptors on endothelial cells and activate them. It is also possible for growth factors from this family to interact with neuropilins, cadhedrins and integrins and heparan sulfate, which could potentiate the effects of VEGFR. VEGF-A is secreted to the greatest extent by endothelial cells but can also be secreted by e.g., thrombocytes, macrophages, dendrocytes, astrocytes, osteoblasts, lymphocytes and tumor cells. It causes expansion and increase of vascular permeability, inhibits apoptosis, enhances proliferation and enhances the recruitment of inflammatory cells—macrophages and granulocytes [24].

VEGF-B has two isoforms VEGF-B167 and VEGF-B186. Both isoforms bind to VEGFR-1. VEGF-B is found mainly in skeletal and heart muscle and in the pancreas. It has a great influence on the proper shaping of the cardiovascular system and the heart muscle itself in the womb. The role of VEGF-B in angiogenesis is believed to be less important, and its most important function under physiological conditions is to enable the survival of smooth muscle cells, neurons, pericytes, myocardial cells and vascular endothelial cells; however, it also aids tumor progression [11,24].

There are four isoforms of PlGF (PlGF-1-4) in humans. PlGF binds to heparan sulfate and the same receptor as VEGF-B, namely VEGFR-1. In addition, PlGF-2 has the ability to bind to NP-1, NP-2 and heparin of the extracellular matrix. The presence of this factor has been found in the trophoblast, the endometrium and in the heart, lungs and skin. It affects angiogenesis, although it does not increase the proliferation or vasodilation by itself—it indirectly displaces VEGF-A from VEGFR-1, due to which VEGF-A can bind to VEGFR-2. Thus, it enhances the action of this factor in pathological conditions, for example in ischemia, inflammation and cancer [11,24].

VEGF-C and VEGF-D are produced from precursors by proteolytic cuts. High expression of VEGF-C is observed in the period of embryonic development during the formation of lymphatic vessels, as well as in adults in the heart, thyroid, ovary, placenta and intestine. It promotes lymphangiogenesis by strongly activating VEGFR-3 receptors and has a lower affinity for VEGFR-2, the activation of which has a weak effect on angiogenesis. Newer sources indicate that it probably also binds to the NP-2 receptor, thus enhancing the activity of VEGFR-2. VEGF-D is most abundant in the lungs of fetuses, but is also found in adults in the muscles, lungs, heart and intestine. It has similar properties to VEGF-C, but unlike it, it only binds to the VEGFR-3 and NP-2 receptor—so it only affects the process of lymphangiogenesis [24].

VEGF receptors (VEGFR-1, VEGFR-2 and VEGFR-3) have seven immunoglobulin-like domains in the extracellular region and a tyrosine kinase domain in the intracellular region. VEGFR-1 and VEGFR-2 are expressed in vascular endothelial cells and hematopoietic stem cells. In addition, VEGFR-1 expression in monocytes and macrophages has also been demonstrated. VEGFR-3 expression has been shown in lymphatic endothelial cells [11]. The binding of VEGF to the extracellular domain leads to the activation of tyrosine kinase in the intracellular domain. VEGFR-1, also known as Flt-1, belongs to the RTK family (receptor tyrosine kinases). This receptor has 10 times higher affinity for VEGF than VEGFR-2 does, which results in its strong influence on the migration of endothelial cells and inflammatory cells in the course of pathological angiogenesis. VEGFR-2, also referred to as KDR, is the dominant receptor also belonging to the RTK family, with a molecular weight of 200–230 kDa. The binding of VEGF to VEGFR-2 leads to the activation of the PLCγ/PKC pathway as well as of the Ras/Raf/ERK MAPK and PI3K/Akt pathways—thus influencing physiological and pathological angiogenesis. It has a certain anti-apoptotic effect and also activates integrins that intensify cell migration. Another of the same family of receptors is VEGFR-3, also known as Flt-4, with a molecular weight of 195 kDa. Its affinity for VEGF-C and VEGF-D is known and also its influence on lymphangiogenesis in both embryonic development and pathological conditions. The signal pathways that stimulate its activation are PKC and Ras as well as Akt/GDP. Activated VEGFR-3 contributes to the proliferation, migration, differentiation and survival of lymphatic endothelial cells. It is also believed to initiate primary lymphedema and is involved in the formation of distant metastases by the lymphatic route [24,25].

### 2.1. Physiological Function of VEGF Family

VEGF family, mainly VEGF-A, plays a key role in the processes of blood vessel formation in embryonic development and in wound healing in adult individuals. It is synthesized by various cell types, including mast cells, smooth muscle cells of blood vessel walls, endothelial cells, monocytes, fibroblasts, macrophages, keratinocytes, eosinophils and T lymphocytes. VEGF undergoes exon splicing, which results in multiple isoforms [26,27]. Wang et al. tried to explain the underlying regulation mechanisms essential to the VEGF signaling pathway. The research revealed four hub genes (*ITBG3*, *PTGS2*, *VEGF* and *MYL9*) that were identified as participants in the VEGF signaling pathway due to their impact on immunosuppression, oxidative stress and endocrine disorder. These findings showed that mainly VEGF and PTGS2 upregulate the VEGF signaling pathway, which results in improving oxygenation [28].

VEGF binds to the extracellular receptor domain, subsequently initiating the activation of the tyrosine kinase enzyme in the intracellular receptor domain, which phosphorylates tyrosine, hence activating certain intracellular signaling pathways. Overall, VEGFR-1 and VEGFR-2 are expressed on vascular endothelial cells, while VEGFR-3 is expressed predominantly on lymphatic endothelial cells. Both VEGF and VEGFRs are also expressed on non-endothelial cells in small amounts. VEGFR-2 is also weakly expressed in hematopoietic cells, retinal progenitor cells, neurons, pancreatic ductal cells and osteoblasts. Interestingly, VEGFR-2 is expressed in early embryonic life as well as VEGFR-3, which is involved in the formation of lymphatic vessels during morphogenesis and in adulthood [24]. In normal tissues, the highest levels of VEGF-A mRNA can be found in the lungs, kidneys, heart and adrenal glands. VEGF-B and PlGF also stimulate angiogenesis in normal tissues, but their activity is much weaker than that of VEGF-A. VEGF-C plays an important role in the development of embryonic lymphangiogenesis. VEGF-D stimulates the growth and migration of lymphatic endothelial cells [11,12]. VEGF proteins have been reported to induce endothelial cell migration and proliferation and to enhance vascular permeability [29].

Angiogenesis, i.e., the formation of new capillaries from the existing ones, is a complex process that is strictly regulated at the molecular level by receptors, growth factors, humoral factors and extracellular matrix proteins. Specific cells participate in angiogenesis with characteristic molecular markers [30]. An important factor driving this process is the state of hypoxia, in which there is the secretion of pro-angiogenic particles, e.g., VEGF, from hypoxic endothelial cells and smooth muscle cells, and other factors such as hypoglycemia, hypertension, lowering of pH and chronic inflammation may be responsible for the hypoxic state. In the classical type of angiogenesis, the permeability of the vessel walls initially increases, and the pericytes lying along them are partially detached due to proteinases. At the same time, hypoxia increases the expression of HIF-1, which results in the secretion of pro-angiogenic factors, including VEGF, FGF-2, PDGF-β, TGF-β, Ang-1, Ang-2 and TNF-α, binding to specific receptors on the endothelial surface and activating signaling pathways. Activated MMPs, mainly MMP-9, MMP-3 and MMP-2, degrade the extracellular matrix and basement membrane, which allows endothelial cells to migrate to the perivascular area toward the VEGF gradient and to proliferate [24,31,32]. Interestingly, MMP-14 and MMP-15 also play an important role in the process of angiogenesis by the degradation of collagen and pro-MMP-2 activation [33].

### 2.2. Pathological Role of VEGF Family

The family of vascular endothelial growth factors influences the process of blood vessel formation in neoplasms, thus promoting the growth of tumors and the formation of metastases. The increase in their expression correlates with the progression of neoplastic disease. Many studies on various types and locations of neoplasms have shown the effect of high expression of VEGF and its receptors on the development of diseases [32]. Interestingly, Kazemi et al. [34] demonstrated that two VEGF splicing isoforms (VEGF121 and VEGF165) are differentially expressed in colorectal cancers and present opposite effects on vessel maturation and tumor growth. Overexpression of VEGF121 results in a decrease of the tumor growth, while VEGF165 increases the expansion of the tumor by promoting the recruitment of smooth muscle cell and vessel maturation in human colorectal cancers. Elevated VEGF expression also occurs in other conditions associated with vascular proliferation, such as atherosclerosis, hemangiomas, liver and kidney diseases, chronic inflammatory diseases, skin and mucosa diseases and retinopathy. There are also states with decreased angiogenesis, in which a decrease in VEGF expression is an important observation, e.g., ischemic disease, coronary artery disease, peripheral vascular disease and leukoencephalopathy and other brain diseases [35].

Factors such as VEGF-A, VEGF-B, PlGF and the VEGFR-1 receptor are involved in the stimulation of pathological angiogenesis, while in the case of lymphangiogenesis VEGF-C, VEGF-D and the VEGFR-3 receptor are the most important contributors [14,15]. Mainly, when a tumor is more than 2 mm in diameter, it cannot grow further based on tissue penetration due to a hypoxic microenvironment of the tumor. Hence the tumor needs the formation of new blood vessels to supply oxygen and nutrients [36]. New vasculature is shaped in and around the tumor to allow it to grow exponentially. Tumor vessels are structurally and functionally abnormal, they are irregularly shaped, have dead ends and are not organized into capillaries, venules and arterioles. This creates suboptimal tumor blood flow resulting in hypoxia and further production of VEGF [27].

Interestingly, recent studies revealed that, in oral cancer, VEGF-A overexpression indicates a poor prognosis, and VEGF-C can be used as a predictive factor in oral squamous cell carcinoma [37]. In esophageal squamous cell carcinoma, VEGF-A can be considered a reference indicator of nodal metastases—its overexpression is significantly associated with tumor infiltration and lymph node involvement [33]. In patients with gastric cancer, studies have shown similar possibilities of using VEGF and confirm that high concentrations of VEGF-C and VEGF-D indicate an unfavorable prognosis of survival [38].

It has also been proven that an increase in PlGF concentration promotes pathological angiogenesis in tumors and inflammatory changes as well as the increase of vascular permeability [14,15]. Stimulation of VEGFR-1 indirectly induces tumor angiogenesis by activating monocytes, macrophages and hematopoietic stem cells. These cells migrate to the tumor and inflammatory lesions to produce VEGF-A, VEGF-C and cytokines, leading to tumor vascularization through VEGFR-2 and VEGFR-3 [11,24].

## 3. Possible Utilities of VEGF Family Members in Colorectal Cancer

### 3.1. VEGF as a Diagnostic Biomarker

Studies present that VEGF expression is overexpressed in most solid tumors [39]. Overexpression of VEGF-D correlates with lymphatic vascular growth and lymphatic metastases. Recent studies suggest that VEGF-D is necessary for the introduction of cancer cells into the lymphatic system, which contributes to the formation of metastases [11,12]. Some researchers have shown that higher preoperative levels of VEGF have been suggested to be associated with the recurrence of colorectal cancer after therapy. Higher expression of VEGF is found as a poor prognostic marker in CRC and poor survival. Moreover, data demonstrate that levels of VEGF are in correlation with tumor stage. Therefore, it suggests a possible role of VEGF in the progression of the disease [40]. The published by Martini et al. [41] showed that quantitative analysis of the VEGF-121 isoform, which is mainly detected in circulating blood, might be a predictor of response to anti-angiogenetic treatment. Interestingly, in recent a publication, it has been presented that in patients with colorectal cancer who underwent chemotherapy VEGF levels tend to increase, but due to the small test group, further investigations are needed [42].

Furthermore, in the work of Celen et al. [43] a positive correlation between VEGF, tumor size and peritumoral vascular invasion was found, and it was even stronger than the correlation of CEA (a tumor marker commonly used in CRC) with those parameters. Additionally, the diagnostic sensitivity of VEGF was higher than that of CEA. It should be stressed that the combination of both markers revealed very high sensitivity for predicting colorectal cancer compared to that of each marker separately. However, despite promising results, this study was performed on small group of patients, and these results need to be confirmed. A similar study was performed by Li et al. [44], where not only the usefulness of VEGF as a neoplastic marker in the course of colorectal cancer was confirmed but a positive correlation of this factor with the size of the tumor was also proven. This may confirm that VEGF is directly secreted in large amounts in the case of hypoxia by the tumor cells themselves. Similar results were obtained in most recent studies conducted by Wang et al. [45]. To the contrary, Abdulla et al. [40] examined the expression of VEGF in tissues of CRC patients. Interestingly, the results have shown upregulation in the early-stage and downregulation in the late-stage of CRC in VEGF expression when compared to normal adjacent tissue. Other studies revealed that examining the miRNA expression, mainly miR-31, might have relevant clinical significance. The high expression of miR-31 was associated with poor differentiation and advanced tumor stage [46]. All the above-mentioned results emphasize the need for monitoring both serum levels and tissue expression of growth factors to fully elucidate their role in patients with CRC.

### 3.2. VEGF as a Therapeutic Target

VEGF proteins are upregulated in colorectal cancer tissue [39]. Studies have shown that anti-VEGF treatment induces vascular regression, therefore inhibiting tumor growth and metastasis [22]. VEGF signaling through VEGFR-2 is the main angiogenic pathway; therefore, the blockage of VEGF/VEGFR-2 signaling is a potential anti-angiogenic strategy for cancer therapy. VEGFR-1 acts as a negative regulator of VEGF-mediated angiogenesis during development and as a stimulator of pathological angiogenesis when activated by PlGF and VEGF-B [11,12].

The first available anti-angiogenic therapy and one of most extensively characterized anti-VEGF agents is bevacizumab. It is humanized monoclonal antibody that binds to VEGF-A isoforms, thereby targeting the tumor microenvironment resulting in inhibition of cell viability, induction of apoptosis and induction of autophagy. It has been shown that synergistic treatment with bevacizumab and chloroquine inhibited tumor growth in tumor model of colorectal cancer cells [39,47]. The studies show that adding bevacizumab to chemotherapy prolongs the survival rates and progression-free period for metastatic colorectal cancer (mCRC). Under bevacizumab treatment, hypoxia and impaired homologous recombination repair (HRR) were detected as well as vascular regression. Tumor hypoxia usually occurs during the anti-angiogenic period and is suspected to play a significant role in anti-angiogenic drug resistance [48]. Nevertheless, the long-term use of bevacizumab has some side effects, e.g., osteonecrosis [36].

Other approved anti-angiogenic agents for metastatic colorectal cancer are regorafenib, ramucyrumab, and ziv-aflibercept [49]. PlGF promotes pathological angiogenesis in tumors by recruiting angiogenic macrophages to tumors; therefore, targeting PlGF could be beneficial in cancer. VEGF-B has limited angiogenic potential, but recently, its role in regulating lipid metabolism in the heart has been found. VEGF-C and VEGF-D induce lymphangiogenesis via VEGFR-3 and have also been shown to stimulate metastasis. The anti-VEGFR-2 antibody inhibits primary and metastatic tumor growth, indicating a key role for VEGFR-2 in tumor angiogenesis [11,12].

A recent Kabel et al. [50] publication reports that topiramate might be an effective adjuvant line of treatment of colon cancer in the future due to its properties of declining tissue VEGF, decreasing serum CEA and positively impacting body weight gain. The research was conducted in an animal model and requires further investigations.

The studies also show that microRNAs (miRNAs), molecules containing around 20–22 nucleotides, might affect most cellular pathways [36]. The induction of miR-125 inhibited the expression of VEGF, because miR-125 directly targets VEGF and represses its expression [51]. Another gene modulator, miR-182-5p, was confirmed to downregulate VEGF-C and VEGF-A as well as VEGF receptor (VEGFR)-2 and VEGFR-3, which results in the inhibition of proliferation and invasion of colon cancer cells. Therefore, it is considered as a potential pathway in CRC patients’ treatment [52]. In addition, in a recent publication, Strippoli et al. [53] reported that some c-MYC-linked miRNAs (miR-31-3p, miR-143 and miR-14) might be the potential target to obtain the reduction of anti-EGFR resistance in metastatic colorectal cancer. Moreover, serum miRNAs might be a predictor of tumor recurrence [52].

Moreover, available data suggest that TMEA (3,3′,4′-trimethylellagic acid), a tannin compound isolated from *Sanguisorba officinalis* L., influences VEGF-related apoptotic pathways, resulting in its anticancer activity [54].

Investigations have shown that extracellular granzyme K (GrK), which belongs to the granzymes family (serine proteases that are stored in the granules of cytotoxic cells), might have an impact on inhibition of angiogenesis by triggering endothelial cells to release soluble VEGFR1 (sVEGFR1), which is a receptor that suppresses angiogenesis by sequestering VEGF-A. The GrK protein is detectable in colorectal cancer tissue, and its levels are in positive correlation with sVEGFR1 protein levels and negative correlation with tumor size and T4 intratumoral angiogenesis. These observations may be a valid target for novel anti-angiogenic therapies in CRC [55].

Interestingly, recent studies demonstrate that baicalein, a bioactive flavonoid, directly binds to toll-like receptor 4 (TLR4), hence reducing VEGF expression, which leads to the inhibition of tumor growth and angiogenesis and reduces the cancer’s metastatic potential [56,57].

It has also been reported that amino acids, especially arginine, have shown potential to inhibit angiogenesis by intervening in the production of VEGF in human colon cancer cells. Arginine can lower the production of VEGF; moreover, supplementation of arginine in the diet resulted in lower VEGF receptor levels in tumors [58].

### 3.3. Relationship between VEGF and Apoptosis

Apoptosis is a natural biological process that allows the body’s cells to deactivate, thus leading to their programmed, controllable destruction. Programmed death has a positive effect on the proper development and homeostasis of cells, while preventing their excessive, harmful proliferation. This process depends on many mechanisms, both intra- and extracellular. Intracellular mechanisms include a genetically defined development program, while extracellular aspects involve endogenous proteins, cytokines and hormones, as well as xenobiotics, radiation, oxidative stress and hypoxia. Any changes in the process of controlled cellular death can result in dysfunctions that lead to different pathological conditions, e.g., cancer development [1,59].

It is important to reveal the relationship between VEGF proteins responsible for angio- and lymphangiogenesis processes and programmed cell death based on the correlation between concentrations of growth factors and proteins with pro-/anti-apoptotic properties (e.g., BAX and BCL-2, respectively). In the intrinsic apoptosis pathway, the BCL-2 family of proteins plays a key role in determining the decision to undergo apoptosis [60,61]. This pathway removes unneeded cells during the development stage but is also activated due to cellular damage or stress [62]. VEGF proteins were assessed to induce the expression of anti-apoptotic proteins such as BCL-2 [29]. Interestingly, Zhang et al. [63] demonstrated that convallotoxin has an impact on both apoptosis and angiogenesis. Convallatoxin downregulates the expression of target genes involved cell survival (e.g., *Bcl-2*), angiogenesis (e.g., *VEGF*), metastasis (e.g., *MMP-9*) and proliferation (e.g., *cyclin D1*). These findings show that convallatoxin promotes apoptosis and inhibits angiogenesis through crosstalk between JAK2/STAT3 (T705) and mTOR/STAT3 (S727) signaling pathways in CRC.

The BAX molecules exist as inactive monomers in an equilibrium between the cytosol and mitochondrial membrane. BAX activation results in the formation of macropores in the outer membrane of mitochondria, which initiates morphological changes associated with apoptosis such as the release of cytochrome c, triggering the activation of the caspase cascade [59,60,62]. The ratio of BCL-2 to BAX is a factor that determines the relative sensitivity or resistance of cancer cells to apoptotic impulses and therapeutic drugs. Decrease of the BCL-2/BAX ratio appears to induce the release of cytochrome C and further activate the mitochondrial-dependent caspase cascade to promote apoptosis [54].

Some studies have shown that implementing an angiogenesis inhibitor that does not have cytotoxic properties to tumor cells can increase tumor cell apoptosis and hinder tumor growth by inhibiting endothelial proliferation and migration and/or by generating endothelial apoptosis. Moreover, oncogene expression and the inhibition of tumor suppressor gene activity can prevent apoptosis of tumor cells [61].

## 4. Conclusions

VEGF proteins are overexpressed in most solid tumors and have a significant impact on pathological angiogenesis, which allows tumor to grow expansively. Blockage of VEGF-related pathways seem to be a valid therapeutic target. Moreover, VEGF proteins were assessed to induce the expression of anti-apoptotic proteins. This induction affects the ratio of BCL-2 to BAX, which may result in an alteration of apoptotic impulses and cancer cells’ resistance to therapeutic drugs. Therefore, studies confirming the role of VEGF family members as tumor markers, therapeutic targets or apoptosis inhibitors are needed not only in tumor detection but also in determining and monitoring subsequent treatment.

## Figures and Tables

**Table 1:** Eighth edition of AJCC TNM classification.

Classification	Definition
T—primary tumor	TX: primary tumor cannot be assessed T0: no evidence of primary tumor Tis: carcinoma in situ, intramucosal carcinoma (involvement of lamina propria with no invasion through muscularis mucosae) T1: tumor invades submucosa (through the muscularis mucosa but not into the muscularis propria) T2: tumor invades muscularis propria T3: tumor invades through the muscularis propria into the pericolorectal tissues T4: tumor invades surrounding tissues T4a: tumor invades through the visceral peritoneum (including gross perforation of the bowel through tumor and continuous invasion of tumor through areas of inflammation to the surface of the visceral peritoneum) T4b: tumor directly invades or adheres to other adjacent organs or structures
N—regional lymph nodes	NX: regional lymph nodes cannot be assessed N0: no regional lymph node metastasis N1: metastasis in 1–3 regional lymph nodes N1a: metastasis in 1 regional lymph node N1b: metastasis in 2–3 regional lymph nodes N1c: no regional lymph nodes are positive, but there are tumor deposits in the subserosa, mesentery or nonperitonealized pericolic or perirectal/mesorectal tissues N2: metastasis in 4 or more regional lymph nodes N2a: metastasis in 4–6 regional lymph nodes N2b: metastasis in 7 or more regional lymph nodes
M—distant metastasis	MX: distant metastasis cannot be assessed M0: no distant metastasis by imaging; no evidence of tumor in other sites or organs M1: distant metastasis M1a: metastasis confined to 1 organ or site without peritoneal metastasis M1b: metastasis to 2 or more organs or sites but without peritoneal metastasis M1c: metastasis to the peritoneal surface is identified alone or with other site or organ metastases

## Data Availability

Not applicable.
